# Duct Stenting in Duct-Dependent Systemic Blood Flow, Past, Present, and Future

**DOI:** 10.1007/s00246-024-03492-y

**Published:** 2024-04-25

**Authors:** Dietmar Schranz

**Affiliations:** 1 Pediatric Cardiology, University Clinic Frankfurt, Frankfurt, Germany; 2 Kinderklinik, Johann-Wolfgang-Goethe University Clinic, Theodor-Storm-Kai 7, 60596 Frankfurt, Germany

**Keywords:** Duct stenting, HLHS, Hybrid approach, Percutaneous stage 1, Pulmonary arterial hypertension, Potts-shunt

## Abstract

Arterial duct stenting, pioneered in the early 1990s for newborns with a duct-dependent pulmonary and systemic circulation, has evolved significantly over the past decades. This progressive technique has led to the development of novel therapeutic strategies, including the Hybrid approach introduced three decades ago, and more recently, a complete transcatheter approach for treating newborns with hypoplastic left heart syndrome (HLHS). Subsequently, the transcatheter method has been extended to bi-ventricular lesions and patients with pulmonary hypertension, establishing a reverse Potts-shunt pathophysiology. Considering current experiences, this review aims to assess the strengths, weaknesses, and complications associated with ductal stenting, which represents a critical component of these complex treatment strategies. Despite advancements, the mortality rate of Norwood and Hybrid stage-1 procedures has plateaued, underscoring the importance of enhancing the quality of life of affected patients as the primary therapeutic goal. The prerequisite is a gentle, almost atraumatic medicine, particularly during the newborn period. It is essential to recognize that both the Hybrid and total transcatheter approaches demand comparable experience to Norwood surgery. Successful outcomes hinge on much more than merely inserting a stent into the duct; they require meticulous attention to detail and comprehensive management strategies.

## Introduction

The roots of cardiovascular interventions can be traced back to the groundbreaking achievements of Andreas Gruentzig, in particular his medical sensation “balloon treatment for heart attack” in 1978. The inception of cardiovascular stents took place in 1986 when Ulrich Sigwart and Jacques Puel successfully implanted the first stents [1]. Carlos Ruiz et al. [2] documented the application of stents for maintaining duct patency in infants awaiting heart transplantation (HTX), while simultaneously, John Gibbs et al. [3] proposed palliating infants with hypoplastic left heart syndrome (HLHS) through ductal stenting combined with bilateral pulmonary banding. Despite initial hopes of providing a less invasive alternative to the Norwood operation, this surgical-interventional procedure proved unsuccessful and was eventually not recommended [4]. However, in Giessen, a variation of this novel surgical-interventional procedure was already developed [5] demonstrating success in reducing institutional mortality rates, and surpassed heart transplantation (HTX) as the primary treatment for neonates with HLHS [6]. The Giessen procedure, designed as a completely percutaneous approach, significantly improved the quality of life for affected newborns and their parents (7–9). In Columbus, the approach was modified into a single surgical procedure, integrating transpulmonary ductal stenting after bilateral pulmonary branch banding (bPAB) during the same open-chest approach, leading to the coining of the term “Hybrid approach” [10, 11]. Since most centers currently use the Hybrid approach solely for high-risk patients [12], the unadjusted mortality rates reported by the Congenital Heart Surgeon’s Society (CHSS) [13] are similar with the long-term survival rates of the Norwood strategy, which plateaued at 60% in newborns with HLHS of common risk. Institutions routinely performing the Hybrid approach for HLHS achieved long-term mortality rates of about 75% [9, 14, 15]. Given the diverse outcome data, the evaluation of “Hybrid stage-1-procedure (S1P)” must consider factors such as the aim, experience, techniques, and available materials.

The Giessen Hybrid S1P was originally conceived as a completely percutaneous approach. However, the realization of the “No Norwood, No Hybrid” dream for newborns with HLHS and variants occurred almost 20 years later [16]. The lack of suitable endoluminal pulmonary flow restrictors (PFRs) for newborns led to the development of the Giessen Hybrid approach, which persists today [9, 17, 18]. This approach involves the performance of short, surgically executed bilateral pulmonary banding (bPAB), followed by percutaneous ductal stenting and atrial septal manipulation, if necessary. Over time, the Hybrid procedure has evolved from an initial rescue therapy to a successful Norwood alternative [9, 14]. Additionally, the Hybrid approach, initially developed for palliating neonates with HLHS, has been extended to bridge patients with hypoplastic left heart complex (HLHC) and borderline left ventricle (BLV), providing time for delayed decision-making or extensive neonatal surgery [18–20]. Ductal stenting, specifically in duct-dependent systemic blood flow, is the primary focus of this article. It consistently maintains duct patency, sustaining systemic circulation wholly or in part for several months and, with re-interventions, for years. The progress in ductal stenting has been marked by experience, knowledge exchange, and technical improvements.

According to the American Heart Association guidelines, and drawing primarily on the experience of American pediatric heart centers, cardiac catheterization and interventions in neonates with HLHS and HLHC are recommended with limitations, typically classified as Class IIb, Level of Evidence C [21].

With almost 30 years of experience in ductal stenting for congenital heart defects involving duct-dependent systemic blood flow, the author of this work emphasizes percutaneous ductal stenting as a component of a two-stage hybrid technique or a complete percutaneous one-stage procedure for palliating newborns with HLHS and HLHC. It is important also to note the significance of ductal stenting in young patients with suprasystemic pulmonary arterial hypertension (PAH) as an alternative to surgical or non-ductal interventional reverse Potts-shunt procedures. However, the details of this alternative are not discussed in this work.

### Univentricular Hearts with Arterial Duct (AD) Dependent Systemic Blood Flow (SBF)

Ductal stenting holds a central, though not exclusive, role in both the Hybrid and total transcatheter approach for treating neonates with HLHS [9, 16]. In both, the surgical/interventional and the complete percutaneous S1P, an unobstructed right-to-left-shunt through the arterial duct (AD) is essential. Duct-diameters of 7 to 9mm are necessary in term neonates to ensure complete systemic blood flow. Additionally, surgical or transcatheter bilateral. pulmonary artery (PA) bands are strategically placed to maintain high central PA blood pressures and ensure an adequate right-to-left shunt through the AD. The interplay between the open duct and banded PA branches aims to safeguard the pulmonary vascular bed from too elevated peripheral pulmonary blood pressures. Simultaneously, it also ensured transpulmonary blood flow for sufficient oxygen uptake at rest and under stressful conditions. Moreover, minimizing a diastolic left-to-right shunt is crucial to avoid pulmonary runoff, preventing low perfusion pressures in vital organs or a low cardiac output. Given a competent pulmonary valve, the adequacy PA-bands can be verified through bilateral systolic-diastolic Doppler-flow patterns. Heart rate control achieved by the use of a selective ß1-receptor blocker supports the complex hemodynamic interaction. This in turn, facilitates optimal body growth by improving of the ratio of consumption to limited supply without jeopardizing the integrity of the pulmonary arteries [22, 23].

### Biventricular Morphology with Duct-Dependent Systemic Blood Flow (SBF)

Neonates with borderline left ventricle or hypoplastic left heart complex are identified based on morphological or dysfunctional criteria. The combination of ductal stenting, bilateral PA-banding and secured restrictive atrial communication proves to be more than an effective alternative to demanding neonatal surgeries [18, 19]. By harnessing the considerable potential for postnatal growth of the left heart structures, Hybrid palliation facilitates cardiac recovery in both morphological and functional aspects. Some patients are afforded the opportunity for biventricular repair within a few weeks or months of birth, steering away from a univentricular strategy [17–19]. In contrast to the neonatal Hybrid approach for HLHS, patients with HLHC typically exhibit only partially duct-dependent systemic blood flow. The interventional and follow-up risks are significantly lower, especially concerning cerebral thromboembolic events or coronary ischemia. With restrictive atrial communication and, consequently, sufficient preload, the essential condition for the growth of the left ventricular is met. Adequate pulmonary artery branch bands ensure systemic blood flow support for most of the lower body.

### Ductal Stenting in Pulmonary Hypertension (Reverse Potts-Shunt Pathophysiology)

Drug-resistant suprasystemic pulmonary hypertension (PH) does not necessarily signify the “end-stage” of a PH or an immediate need for lung or lung-heart transplantation [24]. Return to fetal circulation physiology, in part, or the creation of an Eisenmenger pathophysiology offers an alternative for selected patients [25, 26]. Recanalization of the arterial duct followed by stenting has proven to be an effective therapeutic strategy [27]. When applicable, this ductal resuscitation serves as a straightforward transcatheter alternative to surgical methods [28, 29] or other transcatheter Potts-shunt procedures [30]. The prerequisite is the visible of at least a duct ampulla or even a thread-like duct. The objective of establishing communication between the (left) pulmonary artery (LPA) and descending aorta (DAO) is to relieve the pressure-stressed right ventricle and support systemic circulation without inducing hypoxemia of the upper part of the body.

### Ductal Stenting: Materials and Procedural Considerations

Percutaneous stenting of the arterial duct varies significantly from the transpulmonary procedure [8–10]. Arterial duct stenting is conducted under continuous prostaglandin infusion. Whether it’s a one-stage surgical procedure (Columbus technique) or the Giessen Hybrid technique, the routine first step is the performance of branch pulmonary artery banding (rPAB), especially if the patient isn’t referred with a prostaglandin refractory duct obstruction.

In case of a staged surgical/interventional procedure (Giessen technique), following a median sternotomy for placement of bilateral PABs, percutaneous ductal stenting is performed to complete the S1P. In connection with a total percutaneous S1P, pulmonary flow restrictors (PFR) are initially placed, followed by duct stenting. The entire percutaneous S1P is colloquially referred to as the “DIDI”-approach. This term is coined due to dual intervention of percutaneous placement of PFRs followed by transvenous or transarterial ductal stenting as well as the double intention of achieving “No Norwood, No Hybrid” [16, 31].

The stent material comprises self-expanding Nitinol® or medical steel, commonly used for pre-mounted balloon expandable stents. The choice of stent is contingent on the anatomy of the arterial duct and the duct-aortic junction, considering the presence of significant aortic coarctation either before or after ductal stenting. Transpulmonary duct-stenting (Columbus technique) involves a surgically placed short sheath in the main pulmonary artery, followed by the implantation of a self-expanding or balloon expandable stent in the arterial duct under mono-plane fluoroscopy.

Over nearly 30years experience with ductal stenting in patients with duct-dependent systemic blood flow, and with only one procedural death, the percutaneous route has consistently proven reliable. In patients with HLHS awaiting heart transplantation, ductal stenting was initiated with the intention of providing support up to 6months before HTX, even in an at-home setting. For both, complete transcatheter S1P and the Hybrid approach, percutaneous ductal stenting is preferred:

- To minimize the surgical component of the Hybrid approach, thereby minimizing procedure-related mortality
- To account for the high variability of the arterial duct anatomy and the duct-aortic junction. This area must be analyzed before and after duct stenting. In some cases, patients with residual or even mild aortic coarctation, balloon dilatation may be performed still during prostaglandin infusion before ductal stent placement, provided additional stent placement is not required after ductal stenting. Manipulation of the atrial septum is necessary, or at least warrants investigation, in over 50% of HLHS and variants, particularly in BLV/HLHC candidates.

An obstructed aortic isthmus or significant aortic coarctation can be a critical issue that preclude the Hybrid approach or full percutaneous S1P. Therefore, S1P demands a careful transcatheter procedure with a clear follow-up strategy. One reason why ductal stenting in HLHS newborns is best performed with a bi-plane cardiac catheterization equipment is to allow for near-simultaneous 90° lateral and, crucially, 30° right anterior oblique (RAO) views. Considering the specific univentricular hemodynamics in HLHS patients, we generally prefer to perform ductal stenting in elective, already extubated patients, even after surgical bPAB.

**Fig. 1:**
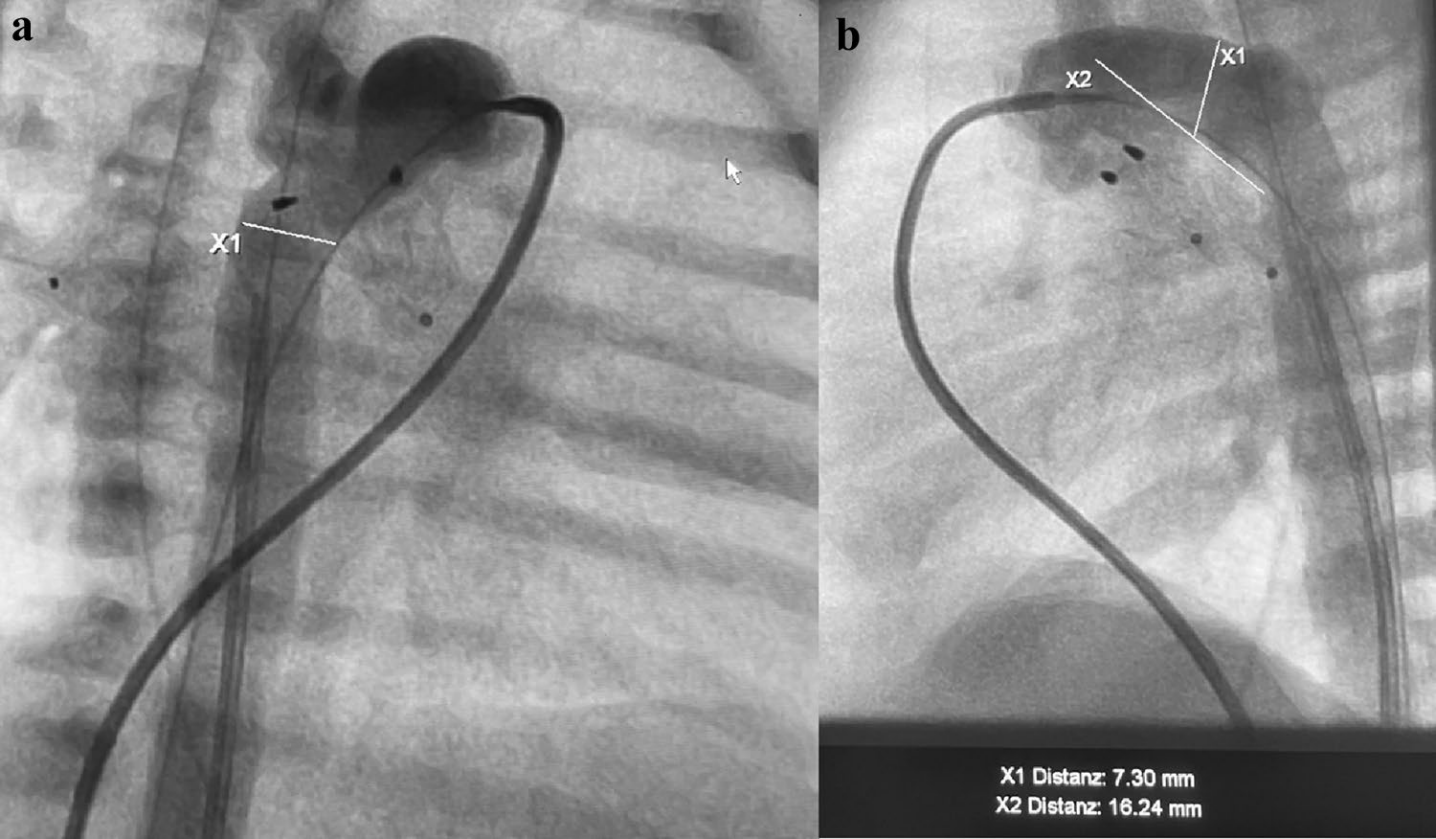
Angiographies to delineate the Arterial Duct**. a** Angiography (30° right anterior oblique projection) is shown to determine the Duct-descending aortic junction. **b** the lateral 90° projection is shown after placement of endoluminal pulmonary flow restrictors (PFRs) with measurements of the duct dimensions prior to ductal stenting

Elective heart catheterization is performed on a spontaneously breathing, contented and euglycemic newborn. A balanced analgesic sedation is administered and medication, if necessary, includes diazepam (0.2–0.5mg/kg), propopfol (0.2–0.5mg/kg), and ketamine (0.5—(1) mg). Diazemules®, diazepam in lipiform, is preferred as the initial medication for awake patients due to its nearly painless application through a peripheral vein. All analgosedatives are administered intravenously in small, separate doses, and repeated if necessary. Analgesic sedation considers the continuous infusion of PGE1, which predisposes to apnea. The local anesthetic in the groin is administered only when the baby is asleep, and femoral vessels are punctured. A 6Fr sheath is inserted if a Rashkind procedure is required; otherwise 4Fr sheaths are sufficient for the percutaneous access to both the femoral vein and artery, if a Sinus-Superflex-DS (SSF-DS, Optimed®, Karlsruhe, Germany) is used. Stent material, either self-expanding Nitinol™ or medical steel, is chosen based on availability and the anatomy of the arterial duct and the morphology of the duct-aortic junction (Fig. 1a, b).

**Fig. 2:**
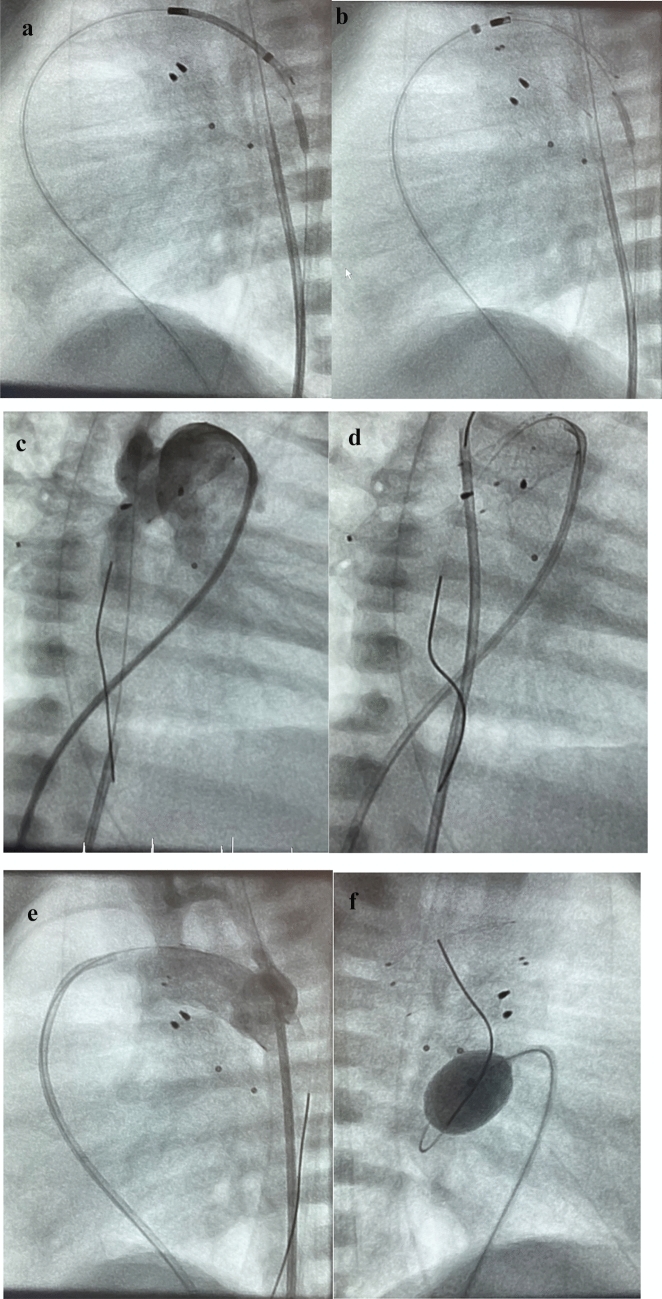
**a** Percutaneous Ductal stenting. Shown is the placement of a Sinus-Superflex-DS stent (SSF-DS, 8 × 20mm) via venous access following endoluminal pulmonary artery branch banding (pulmonary flow restrictor, PFR) in a newborn with HLHS (hypoplastic left heart syndrome; mitral stenosis and aortic valve atresia). **a** Shows the beginning of the SSF-DS expanding in the arterial duct (AD) in a lateral 90° projection; a multipurpose catheter with guidewire placed in the descending aorta (DAO) and PFRs in the left (LPA) and right pulmonary artery (RPA) are good markers for accurate stent placement. **b** shows the fully expanded SSF-DS with the relationship of the SSF-DS to the upper PFR positioned in the LPA **c** shows an angiography by hand-injection through a re-placed 4Fr right Judkins-catheter (JR) in right anterior oblique (RAO) 30° projection. **d** is intended to demonstrate the importance of a re-evaluation of the AD-DAO-junction, in which a multi-purpose (MP) catheter positioned in the DAO passes over the ductal-via a guiding wire or, if necessary, through the struts of the AD-stent is carefully brought forward into the descending aortic arch (AOA) to detect a possible pressure gradient and to rule out a residual coarctation (CoA) angiographically (**e**). **f** shown is the lateral 90° projection after placement of PFR’s and duct-stenting for finalization of the percutaneous stage-1-procedure (S1P) by a Rashkind procedure. The Rashkind balloon is just inflated before the atrial septum is ruptured to achieve an unrestricted atrial communication

In the late nineties, various types of balloon-expandable stents were used for AD stenting [32]. Since 2006, self-expandable nitinol stents have been preferred, initially the closed-cell designed Sinus Repro stent®, which, however, had to be inserted through a 5F sheath (OptiMed, Karlsruhe, Germany). Since 2010, the CE-marked Sinus-SuperFlex-DS™ has been the preferred self-expandable nitinol stent for duct-stenting in newborns with unobstructed duct-dependent systemic blood-flow. This stent design allows for easy antegrade and retrograde stent implantation in the AD without the need for a long guiding sheath, providing advantages such as MRI compatibility and minimal impact on hemodynamics [32]. The SSF-DS is available in diameters (4, 5, 6 mm not useful in duct-dependent systemic blood flow) 7, 8, 9, and 10mm and partly in lengths of 12, 15, 18, 20, and 24 mm. The stent is supplied preloaded in a 4F delivery system. The delivery system is available in 85 cm length. The guidewire lumen is compatible with 0.018″ wires. Stent deployment is controlled by a two-hand over-the-wire pull back system. For better fluoroscopic visibility, the stents have three radiopaque tantalum markings at each stent end (Fig. 2a).

Patient-specific risk–benefit assessment is recommended before ductal stenting, especially when using SSF-DS (Fig. 2b).

Diagnostic reasons warrant the placement of an 4Fr MP-catheter within the DAO for precise stent deployment both as a marker and for short-hand-injections of contrast medium during the procedure (Fig. 2a–f).

**Fig. 3:**
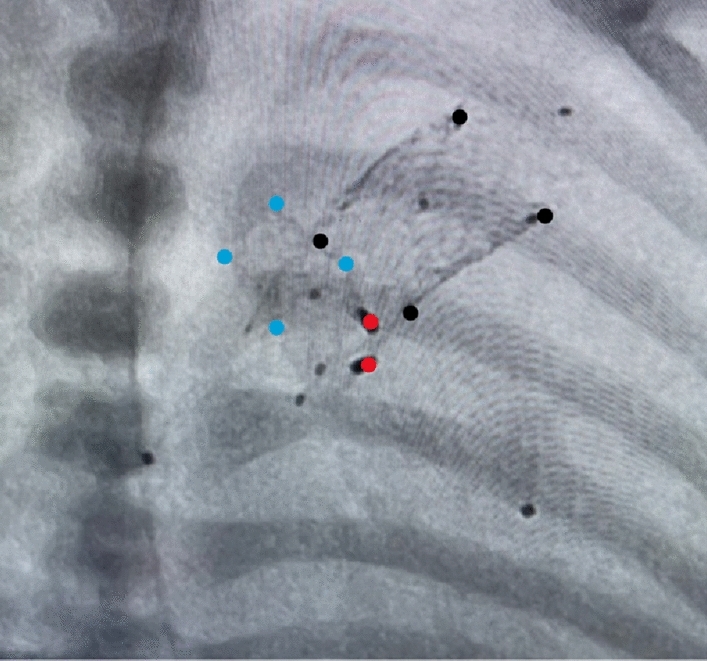
Final Stage 1 procedure (S1P). Shown is the definitive S1P in another neonate with HLHS (aortic and mitral atresia), consisting of bilateral pulmonary flow restrictors (PFR) and two ductal stents (first stent is indicated by black markers). The second stent was placed telescopically to treat significant CoA when PGE1-infusion was stopped two days after initially uneventful S1P. An obstruction occurred distal of the originally placed ductal stent. Furthermore, a significant retrograde gradient was evident after placement of the second ductal stent. The significant CoA required a coronary stent placed through the struts of the second SSF-DS. The blue markers indicate the expanded coronary stent (5 × 8mm), the red markers the proximal ends of the PFRs placed within the LPA and RPA

In case of retrograde ductal stenting via arterial access, a 4Fr right Judkins (RJ) catheter has to be placed at the pulmonary side of the duct to similar reasons and particularly in less experienced hands. Continuing prostaglandin infusion during and after the transcatheter procedure, a hallmark of using SSF-DS, reduces the initial risk of duct spasm and potential foreign body reaction. The SSF-DS design enables uninterrupted blood flow through the duct during stent expansion. The risk of stent embolization is reduced by its open-cell design and careful consideration of stent diameter. The intended stent diameter is 1 to 2mm larger than the smallest diameter of the arterial duct. Stent embolization in the direction of the descending aorta (DAO) can be avoided by choosing a stent diameter larger than the diameter of the DAO. In cases requiring a significant diameter expansion of the AD, balloon-expanding stents are preferred. The length of the stent or telescopically placed stents should cover the entire suspected duct tissue to avoid obstruction post-prostaglandin discontinuation (Fig. 3).

Patient-specific anatomy analysis is crucial, with unclear anatomy making the procedure challenging. In some cases, a two-stage procedure is preferred, particularly in HLHS newborns, to minimize life-threatening complications associated to potential ductal tissue-related vascular obstruction (Fig. 3). Continued PGE1-infusion (5ng/kg/min), together with continuous heparin infusion (300U/kg/24h) offers antithrombotic protection and guards against initial aortic isthmus obstruction. This approach also hypothesizes a potential reduction in stent hyperproliferation. Both drugs are administered until the initiation of oral anti-aggregation treatment with clopidogrel (0.2mg/kg daily dosage) and in part combined with acetylic-salicylic acid (1-2mg/kg/day) in a dosage to block platelet thromboxane production, but not endothelial prostacyclin (see follow-up). A detailed follow-up strategy, including Doppler-flow analysis is essential, especially in patients with narrowed aortic isthmus if not a significant aortic coarctation is already treated by stent placement. Therefore, additional transcatheter treatments may be required based on post S1P analysis. It has to be noted, that thrombus formation within the duct is a rare event, but the general danger of thrombo-embolic events through the right-left-shunting stented duct remains with potential consequences of strokes or coronary insults.

#### Follow-Up Management in Newborns with HLHS/HLHC

After the transcatheter procedure, all electively treated patients are transferred back to the intermediate care ward, where the mother awaits her child in a mother–child unit. In addition to maternal monitoring and care, children are continuously monitored for heart and respiratory rates, pulse oximetric oxygen saturation, and intermittent blood pressure measurements. Following percutaneous S1P in newborns with HLHS, systolic and diastolic blood pressures on the right arm (reflection of coronary and cerebral perfusion) and on the leg that not has been used for catheterization is measured several times a day while the child is sleeping. Early exclusion of pre- or post-ductal coarctation is highly important. Unremarkable transthoracic (TTE) echocardiographic examination, specifically excluding duct obstruction or aortic coarctation, with acceptable retrograde perfusion of the aortic arch, including the cerebral arteries, and perfusion of the descending aorta including the celiac trunk is required to stop PGE1-infusion. Following percutaneous S1P, when good oral feeding is achieved, an overlap of oral clopidogrel and a really low dose acetylsalicylic acid (ASA) is used. The cyclo-oxygenase inhibitor ASA is administered with the hypothesis that the very low dosage (1-(2) mg/kg once per day) together with a neonatal dosage of clopidogrel only achieves an antiaggregative platelet effect. Endothelial prostacyclin production should not be additionally blocked or induce an obstructive duct effect, which may be is the case with a higher ASA dosage.

The preprocedural daily administration of the highly specific ß1-adrenergic receptor blocker bisoprolol at a dosage of 0.1mg/kg/day is continued after the percutaneous intervention, aiming for heart rate of less than 120 (130)/min during sleep for a HLHS newborns at term. In case of a post-procedural state of still excitement not caused by hunger (glucose level!), clonidine is given orally 4-(6) times daily at a dosage of 0.5-1µg/kg, so that clonidine or dexmedetomidine infusion can be avoided.

The application of a long-acting ACE-inhibitor is only applied, but if used, almost always in combination with a ß-blocker, if no residual aortic obstruction is evident and the systemic vascular resistance needs to be further reduced without endangering the perfusion pressures of vital organs. Spironolactone is additionally used in low, non-diuretic dosage under the hypothesis to influence myocardial hypertrophy with likely consequent diastolic dysfunction [34, 35].

It is additionally noted that in general, patients with congenital shunt defects, including HLHS or patients with cardiomyopathy in whom pulmonary artery banding is indicated, diuretic treatment or the administration of catecholamines for inotropic properties is usually not indicated, but is rather counterproductive or even dangerous.

**Fig. 4:**
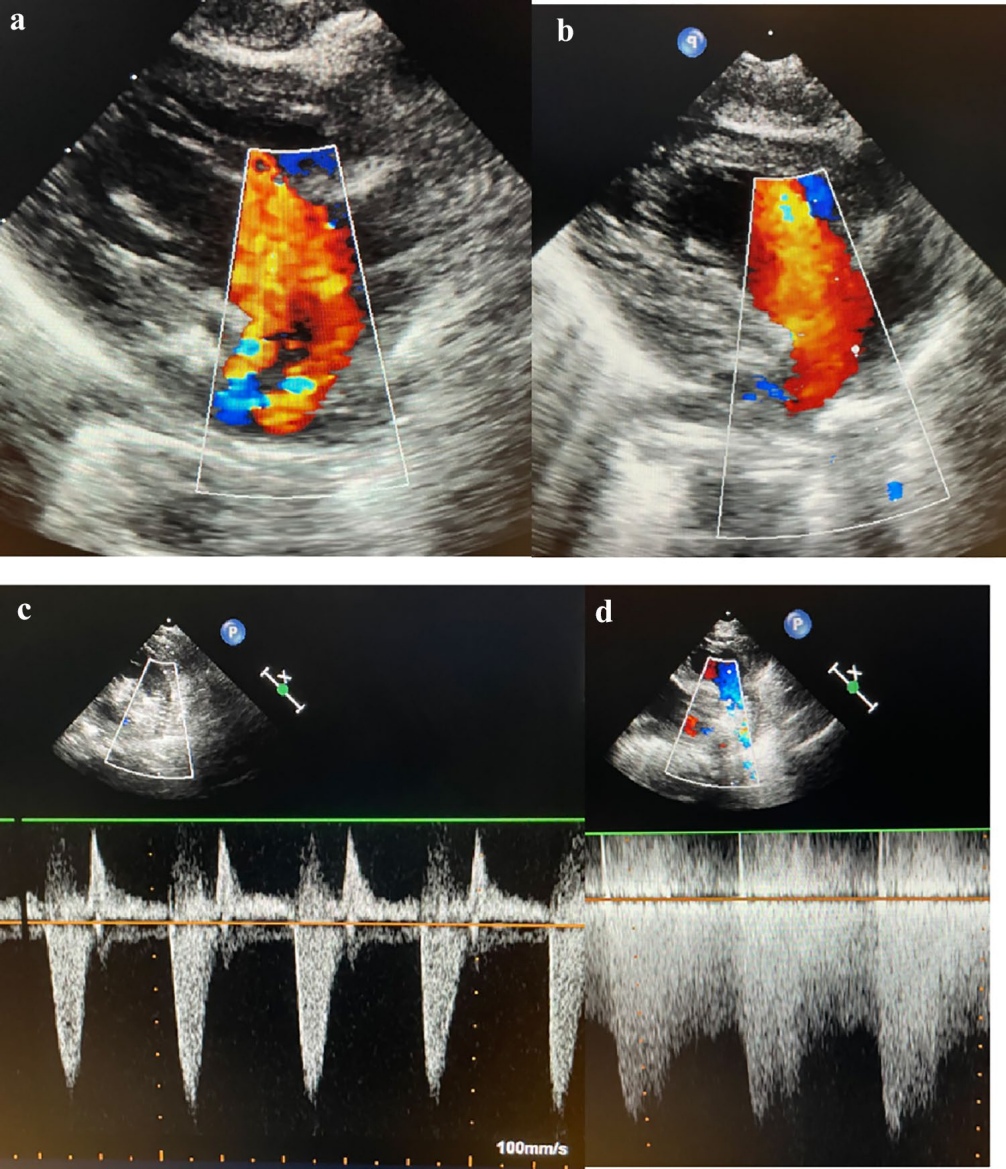
Percutaneous S1P: echocardiographic documentation. **a** The interatrial communication of a multiperforated atrial septum is shown with two red-colored left-to-right jets. The diminished left ventricle with a hypoplastic mitral valve is adumbrated. **b** An unobstructed atrial communication after Rashkind procedure is shown with laminar red-colored flow from left atrium through ruptured atrial septum through the tricuspid valve. **c** CW-Doppler is shown with adequate systolic right-to-left through the stented arterial duct with an acceptable diastolic left-to-right shunt component due to PFRs in the pulmonary artery branches. **d** Shows a systolic-diastolic CW-Doppler pattern corresponding to highly efficient endoluminal banding of the LPA by a PFR

After discharge, routine but closer follow-up visits are scheduled every eight to ten days. Assessments include detailed short-term parental reports, clinical evaluations of heart rate, saturation, blood pressure measurements, and TTE (Fig. 4a–d).

Routine re-catheterization is not necessarily indicated, but correcting interventional procedures can become necessary.

Comprehensive stage II (cS2P) is already planned at the day of birth and when a successful S1P has been achieved. Considering the exponential endothelial or total vascular proliferation of all artificially affected vascular area with its associated hemodynamic risks, we schedule the cS2P exactly 4 months after birth. This time strategy seems to be much more important as more foreign bodies (several stents, pulmonary flow restrictors) are used or anatomical consideration such as an ascending aorta of less than 2mm, tricuspid valve regurgitation or intermittent ischemic attacks, limited retrograde aortic arch flow are verifiable. Bridging to deferred Norwood procedure is necessary in few selected patients.

#### Complications Associated with the Hybrid Approach

Avoiding complications requires a well-prepared team, not only for a sufficient Norwood program but also for a successful Hybrid approach or full percutaneous S1P. High-risk patients with low birth weight, a tiny ascending aorta (AAO) with golf hole connection at the aortic arch, or existing myocardial insufficiency with tricuspid valve regurgitation and an overly restrictive atrial septum present challenging starting conditions for establishing a Hybrid approach.

Induction of anesthesia with relaxation, intubation, and positive pressure ventilation can be fatal due to their negative effects on balanced hemodynamics.

Our routine thromboprophylaxis with heparin in combination with prostaglandin results in very rare stent thrombosis in duct-dependent systemic blood flow. However, a high risk of thromboembolic events remains not only until comprehensive S2P but even to Fontan circulation, if performed with a fenestration. In our initial Hybrid series of 156 patients [8], which included the initial learning curve [17], one high-risk patient died on the catheter table when only premounted balloon expanding stents were used for duct stenting. The non-resuscitable patient had an extremely small AAO of less than 1mm and died due to disruption of systemic blood flow during stent expansion. Additionally, AAO compression by balloon expansion of the stent was discussed [18].

Acute complications such as stent slippage, malposition or embolization can be reduced, but not completely ruled out, by considering the above-described measures and recommendations. Self-expanding, open-cell designed stents, such as the SSF-DS deliverable through a 4Fr short sheath, have enormous advantages in preventing stent embolization when stenting an unobstructed duct. In case of embolization, it can mostly be revised using interventional techniques. Disadvantages due to the weakness of the stent material-related radial force may make it unsuitable for some applications. Further complications could be associated with stent collapse or kinking, which can be avoided by using balloon expanding stents instead or by placing a second stent with or without previous post-dilatation of the first stent.

The interstage is a vulnerable period with comparable adverse events to those following the Norwood S1P. With S1P mortality of less than 3% and an interstage mortality of 5 -10%, close follow-up examinations and sufficient parental education are crucial to detecting early duct-stent and retrograde aortic obstructions or restriction of the atrial communication. Coronary and Cerebral ischemia by retrograde blood-flow obstruction poses the highest risk in the interstage. The highest risk is associated with residual non-covered duct-tissue or preformed proximal or distal aortic coarctation in combination with aortic atresia and extreme small AAO.

Preventive or treating CoA stenting does not guarantee an unobstructed course during the interstage. Any stent material disposed to neointima proliferation with tissue ingrowth through the stent struts may necessitate re-dilatation or placement of a second stent. Limitations of re-interventions should be considered in case of initial stent-placement through the struts of the primarily placed ductal stent (Fig. 3). Overall, a sophisticated follow-up observation is crucial for successful transcatheter concept. The Hybrid or DIDI approach is more than placing a stent in the duct after surgical or transcatheter bilateral branch banding. This underscores the need for a percutaneous duct stenting with pre-and post-interventional re-check in a holistic matter.

## Conclusion

Ductal stenting in neonates with duct-dependent systemic circulation has become a routine transcatheter procedure. While it is technically relatively straightforward to place a stent in the duct, its precision remains a challenge. The risks and pitfalls are associated with the complexity of the left heart disease and the variable morphology of the duct-DAO junction. Moreover, the ductal stent approach is typically one piece in the mosaic of an orchestra of therapeutic measures. Considering that the long-term survival rate for Norwood or Hybrid procedures plateaus at 60% to 75%, the therapeutic focus must shift toward improving the quality of life while self-guarding the genetic endowment [36]. The combination of surgical pulmonary artery branch banding and ductal stenting, known as the Hybrid approach, was the attempt reducing mortality and enhancing the quality of life. The integration of endovascular pulmonary branch banding and transcatheter ductal stenting (DIDI-approach) represents the next step in abandoning surgical procedure with anesthesia and intensive care during the neonatal period, transitioning instead to later infancy. However, the current experience with the DIDI-approach remains limited. Both term and preterm infants with HLHS and HLHC have already undergone successful palliation, along with young infants with other congenital heart defects and syndromes associated with pulmonary overcirculation through left-to-right shunt defects [16, 36–40]. To the best of our knowledge, the first patients treated for the first time with the “DIDI” approach are all in a good condition [16]. Three children with HLHS and one with a different complex single ventricle physiology received successful Fontan completion after comprehensive stage II procedure. One child received biventricular repaired during infancy, and another patient, initially listed after S1P, is now living with heart transplant.

It is important to emphasize that all patients underwent percutaneous S1P only under sedation with spontaneous breathing while asleep, including all elective patients afterward. This gentle treatment regime is considered essential to improve the quality of life for the affected newborns and young infants with HLHS and variants [33]. The decision whether to use potentially dangerous anesthesia for percutaneous S1P should not be left solely to the anesthetist. Balanced, low-threshold sedation can be learned and implemented irrespective of the locality.

Normal postnatal mother–child bonding is achievable not only in electively treated babies. It is evident that the novel technology brings significant benefits, particularly in high-risk patients such as premature or low-weight babies, those with syndrome-related shunt defects, as well as newborns admitted with acute heart failure or cardiogenic shock caused by pulmonary run-off or duct obstruction. What is now essential is to gather evidence that this strategy will lead to the desired success for these children in their lives, especially when a substantial cohort of patients is treated. The Hybrid approach can theoretically be replaced by full percutaneous S1P even in neonates with severe congenital aortic valve stenosis, left ventricular heart failure, and severe mitral valve regurgitation. Subsequent destination, as bi-ventricular repair, univentricular strategies, or even heart transplantation, can be made possible through percutaneous S1P. However, caution should be exercised with any new technology or clinical approach, simply because experience is still limited and a learning curve is required. Ductal stenting is just one part of a complex treatment. Until the definitive operation, it must always be taken into account that the pathophysiology of a duct with right-left shunt remains. This makes the acute treatment and also the interstage course vulnerable.
